# eIF4B is a convergent target and critical effector of oncogenic Pim and PI3K/Akt/mTOR signaling pathways in Abl transformants

**DOI:** 10.18632/oncotarget.7164

**Published:** 2016-02-03

**Authors:** Ke Chen, Jianling Yang, Jianning Li, Xuefei Wang, Yuhai Chen, Shile Huang, Ji-Long Chen

**Affiliations:** ^1^ CAS Key Laboratory of Pathogenic Microbiology and Immunology, Institute of Microbiology, Chinese Academy of Sciences (CAS), Beijing 100101, China; ^2^ College of Animal Sciences, Fujian Agriculture and Forestry University, Fuzhou 350002, China; ^3^ Key Laboratory of Immune Mechanism and Intervention on Serious Disease in Hebei Province, Department of Immunology, Hebei Medical University, Shijiazhuang 050017, China; ^4^ Department of Biochemistry and Molecular Biology, Louisiana State University Health Sciences Center, Shreveport, LA 71130, USA

**Keywords:** Abl oncogene, eIF4B, Pim kinase, AKT, tumorigenesis

## Abstract

Activation of eIF4B correlates with Abl-mediated cellular transformation, but the precise mechanisms are largely unknown. Here we show that eIF4B is a convergent substrate of JAK/STAT/Pim and PI3K/Akt/mTOR pathways in Abl transformants. Both pathways phosphorylated eIF4B in Abl-transformed cells, and such redundant regulation was responsible for the limited effect of single inhibitor on Abl oncogenicity. Persistent inhibition of one signaling pathway induced the activation of the other pathway and thereby restored the phosphorylation levels of eIF4B. Simultaneous inhibition of the two pathways impaired eIF4B phosphorylation more effectively, and synergistically induced apoptosis in Abl transformed cells and inhibited the growth of engrafted tumors in nude mice. Similarly, the survival of Abl transformants exhibited a higher sensitivity to the pharmacological inhibition, when combined with the shRNA-based silence of the other pathway. Interestingly, such synergy was dependent on the phosphorylation status of eIF4B on Ser422, as overexpression of eIF4B phosphomimetic mutant S422E in the transformants greatly attenuated the synergistic effects of these inhibitors on Abl oncogenicity. In contrast, eIF4B knockdown sensitized Abl transformants to undergo apoptosis induced by the combined blockage. Collectively, the results indicate that eIF4B integrates the signals from Pim and PI3K/Akt/mTOR pathways in Abl-expressing leukemic cells, and is a promising therapeutic target for such cancers.

## INTRODUCTION

Abl oncoproteins, including v-Abl, Bcr-Abl and Tel-Abl, are activated non-receptor tyrosine kinases that are associated with various malignant transformations of hematopoietic cells [1–3]. v-Abl, the retrovirally transduced product of Abl, contributes to murine pre-B cell malignant transformation [1]. Bcr-Abl, resulting from chimeric chromosome translocation, is the major cause of human chronic myelogenous leukemia (CML) [2]. Expression of Abl kinase constitutively activates multiple signaling pathways, including Janus family of kinase/signal transducers and activators of transcription (JAK/STAT)-dependent Pim signaling and PI3K/Akt/mTOR that are critically involved in the regulation of cell proliferation and survival [1, 4–6]. Despite extensive studies, the precise mechanism underlying Abl-induced hematopoietic malignances is not fully elucidated [4–7]. In particular, the relationship between JAK/STAT/Pim and PI3K/Akt/mTOR signaling in Abl transformants is poorly understood.

Growing evidence suggests that alterations in protein expression occur in various cancers [8, 9]. However, the precise pathogenic processes remain to be unveiled. It has been shown that Abl oncoproteins not only activate the transcription of numerous genes involved in cell proliferation or survival, but also dysregulate the mRNA translation program of such genes [9–11]. Under normal circumstances, the translation processes are accomplished by coordinated events and precisely controlled by a variety of regulatory elements in a signal dependent pattern [12–14]. Among the steps of mRNA translation, the control of translation rate is preferentially exerted at the initiation phase, which is crucial for the specific expression of genes important for cell growth, proliferation, and survival [12–14].

For eukaryotic translation initiation, an essential event is the assembly of the translation pre-initiation complex (PIC) [13, 14], which contains various eukaryotic translation initiation factors (eIFs) [13, 15]. In particular, eIF4E, eIF4G and eIF4A constitute a complex named eIF4F that is critical to mRNA translation initiation [13–15]. The ribosome recruitment to the 5′ ends of eukaryotic mRNAs is mediated by the eIF4F complex in which eIF4A, an ATP-dependent RNA helicase, melts the secondary structure of mRNA for the subsequent scanning [16]. eIF4B is thought to stimulate eIF4F activity through enhancing the eIF4A RNA helicase activity in unwinding secondary structures in the 5′ untranslated region (5′UTR) of the mRNA [13, 15, 16]. Therefore, eIF4B is a key component in control of the major rate-limiting step of protein synthesis.

eIF4B is involved in translation of numerous proliferative or anti-apoptotic mRNAs with highly structured 5′UTR and subsequently affect cell growth and survival [17, 18]. Abnormal protein levels or phosphorylation levels of eIF4B are associated with a variety of tumors including Abl-positive CML, T-cell lymphoblastic leukemia, breast cancer, Kaposi sarcoma, and diffuse large B-cell lymphoma [18–21]. Recently, we have observed that eIF4B is highly phosphorylated in Abl transformants and promotes both Bcr-Abl and v-Abl-mediated cellular transformation [4]. Remarkably, eIF4B knockdown impairs the transforming efficiency by Abl oncoproteins [4].

The activity of eIF4B relies on its phosphorylation status [22, 23]. It has been identified that Ser422 is the main phosphorylation site of eIF4B targeted by several signaling cascades, and the phosphorylation level of Ser422 is important for its role in tumorigenesis [22–24]. Recently, we have identified eIF4B as a substrate for both Pim-1 and Pim-2 kinases in Abl transformants and demonstrated that Ser422 is a critical residue in eIF4B that can be regulated by Abl-dependent signaling [4]. Besides, eIF4B can also be phosphorylated by AGC protein kinase family [22–25]. For example, ribosomal S6 kinases (S6K1 and S6K2) and Akt can phosphorylate eIF4B on Ser422 residue [24]. Both S6K and Akt are key components of PI3K/Akt/mTOR pathway, and activated by phosphatidylinositol-dependent kinase 1 (PDK1), a downstream molecule of PI3K. In PDK1-null cells, eIF4B Ser422 phosphorylation is abrogated [22]. Additionally, it has been demonstrated that p90 ribosomal protein S6 kinase (RSK) can also mediate eIF4B Ser422 phosphorylation in response to growth factor stimulation [22]. Together, these data suggest that eIF4B may be a common substrate of several crucial signaling pathways involved in cell proliferation and survival. However, it is still unclear how eIF4B mediates the effects of these kinases on cellular transformation, and little information is available about whether there exists an interaction between these signaling pathways in Abl transformants.

In this study, we found that eIF4B was a common substrate of oncogenic Pim and PI3K/Akt/mTOR pathways in Abl transformants, and such redundant activation of eIF4B is responsible for the compromised effect of pharmacological inhibition of single pathway on Abl tumorigenesis. By contrast, combined treatment to block both pathways caused a deeper inhibition of eIF4B phosphorylation, induced more apoptosis in Abl transformed cells, and synergistically inhibited K562 xenograft growth in nude mice. Overexpression of eIF4B phosphomimetic mutant S422E remarkably attenuated the synergistic effects of these inhibitors on suppression of Abl oncogenicity. These results establish a key role of eIF4B in Abl tumorigenesis by integrating the functions of oncogenic Pim and Akt signaling pathways, and highlight eIF4B as a novel therapeutic target for Abl positive cancers.

## RESULTS

### Phosphorylation of eIF4B is regulated by PI3K/Akt/mTOR pathway in v-Abl and Bcr-Abl transformants

**Figure 1:**
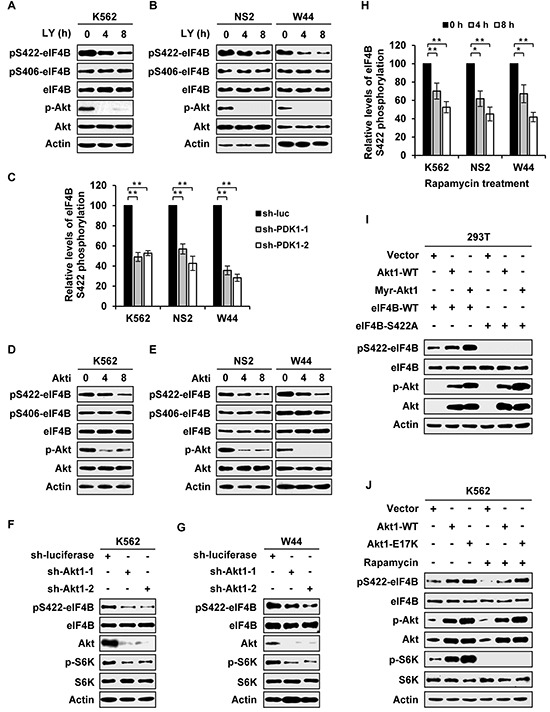
eIF4B phosphorylation is regulated by PI3K/Akt/mTOR pathway in Abl transformants **A.** Bcr-Abl transformed cells (K562) were treated with LY294002 (10 μM) for indicated time. Whole cell lysates were prepared and examined for eIF4B Ser422 or Ser406 phosphorylation levels by Western blotting. **B.** v-Abl transformed cells (NS2 and W44) were treated with LY294002 (5 μM) for indicated time. The eIF4B phosphorylation was analyzed as described in A. **C.** eIF4B Ser422 phosphorylation levels in Supplementary Figure 1B and 1C were quantitated by densitometry and normalized to total protein levels. The eIF4B S422 phosphorylation levels of Abl transformants expressing sh-luciferase were set to 100%. Plotted are results from three independent experiments. Error bars represent SEM, *n* = 3 (**P < 0.01). **D.** and **E.** Bcr-Abl^+^ cells (D) or v-Abl^+^ cells (E) were treated with 4 μM (D) or 1.5 μM (E) Akti-1/2 for indicated time. Analysis of eIF4B phosphorylation were performed as described in A. **F** and **G.** K562 (F) or W44 (G) cells expressing luciferase-specific shRNA or Akt1-specific shRNAs were analyzed by Western blotting with indicated antibodies. **H.** eIF4B Ser422 phosphorylation levels in Supplementary Figure 1H and 1I were quantitated by densitometry and normalized to total protein levels. The levels of eIF4B S422 phosphorylation were set to 100% at 0 hour. Plotted are results from three independent experiments. Error bars represent SEM, *n* = 3 (*P < 0.05, **P < 0.01). **I.** eIF4B-WT or S422A mutant was co-transfected with empty vector, Akt1-WT, or Myr-Akt1 in 293T cells. Total proteins were extracted and analyzed for eIF4B S422 phosphorylation by Western blotting. **J.** K562 cells overexpressing Akt1-WT, Akt1-E17K, or control were treated with or without rapamycin (10 μM) for 4h and analyzed as described in Figure 1I with indicated antibodies.

Recently, we observed that phosphorylation of eIF4B was not completely abolished by disrupting the expression of Pim-1 and Pim-2, implying that there exists other Ser/Thr kinase(s) responsible for eIF4B phosphorylation in Abl-expressing leukemic cells [4]. To test this possibility, we employed several pharmacological inhibitors and shRNAs to determine the involvement of PI3K/Akt/mTOR pathway in this event. Indeed, the phosphorylation levels of eIF4B Ser422 in Bcr-Abl (K562) or v-Abl (NS2 and W44) positive leukemic cells decreased significantly in response to treatment with PI3K inhibitor LY294002 (Figure 1A and 1B and Supplementary Figure S1A). Our previous studies revealed that STAT/Pim-1 and mTOR/S6K pathways phosphorylate eIF4B mainly on Ser422 in Abl transformants [4], and it was shown that inhibition of Akt had no effect on eIF4B Ser406 phosphorylation in A14 cells [23]. To verify whether PI3K/Akt/mTOR pathway contributes to eIF4B Ser406 phosphorylation in Abl transformants, we examined the phosphorylation of eIF4B Ser406 in response to LY294002 treatment. In contrast to Ser422, inhibition of PI3K had no significant effect on Ser406 phosphorylation in both Bcr-Abl and v-Abl transformants (Figure 1A and 1B). To confirm this finding, we generated stable K562, NS2 and W44 cells expressing shRNAs specifically targeting PDK1, another downstream effector of PI3K and an upstream regulator of Akt/S6K [22]. As predicted, knockdown of PDK1 inactivated Akt and S6K, and resulted in remarkable suppression of eIF4B S422 phosphorylation but had little effect on Ser406 phosphorylation (Supplementary Figure S1B and S1C and Figure 1C).

Furthermore, we observed that the phosphorylation level of eIF4B Ser422 was obviously reduced by treatment with Akti-1/2, an isozyme-selective inhibitor of Akt (Figure 1D and 1E and Supplementary Figure S1D). Consistent with previous reports [23], Ser406 was little affected by Akti-1/2 treatment (Figure 1D and 1E). To directly demonstrate the involvement of Akt in eIF4B phosphorylation, we generated stable Abl-expressing cells expressing shRNA to Akt1. As expected, the phosphorylation level of eIF4B on Ser422, not on Ser406, was markedly decreased by silencing Akt1 in both Bcr-Abl and v-Abl positive cells (Figure 1F and 1G and Supplementary Figure S1E-S1G).

In addition, rapamycin was employed to determine the functional relevance of mTOR, a downstream effector of PI3K/Akt pathway, in eIF4B phosphorylation. Interestingly, treatment of cells with rapamycin also significantly impaired the eIF4B phosphorylation on Ser422 but had no obvious effect on Ser406 phosphorylation (Supplementary Figure S1H and S1I and Figure S1H). Because a previous study using an *in vitro* kinase assay showed that Akt can directly phosphorylate eIF4B on Ser422 [23], we asked whether Akt-dependent phosphorylation of eIF4B totally required activation of mTOR/S6K signaling in Abl transformants. To address this issue, eIF4B wild-type or its Ser422Ala mutant was co-transfected with either Akt wild-type or its active form in 293T and K562 cells. We found that forced expression of Akt, especially its active mutants, markedly elevated the eIF4B phosphorylation (Figure 1I). Treatment with rapamycin potently suppressed eIF4B phosphorylation in the control cells transfected with empty vector, but failed to completely block eIF4B Ser422 phosphorylation induced by overexpression of either wild-type or active form of Akt (Figure 1J and Supplementary Figure S1J). Together, these data suggest that PI3K/Akt pathway regulates eIF4B Ser422 phosphorylation in both mTOR/S6K-dependent and -independent manners in Abl transformants.

### Persistent inhibition of one signaling pathway causes enhanced activation of the other pathway and thereby restores eIF4B Ser422 phosphorylation in Abl transformants

**Figure 2:**
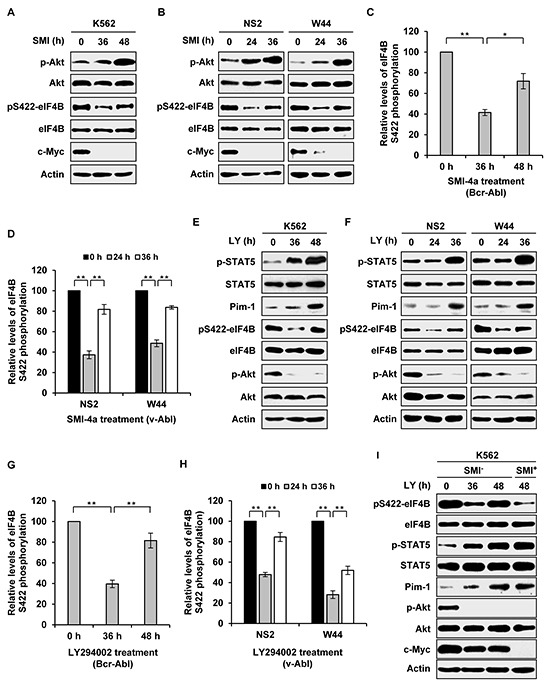
Long time inhibition of one signaling activates the other pathway and restores eIF4B Ser422 phosphorylation **A.** K562 cells were treated with 5 μM SMI-4a for indicated time. Examination of eIF4B and Akt phosphorylation was performed with indicated antibodies. **B.** NS2 and W44 cells were treated with 2 μM SMI-4a for indicated time. The phosphorylation of eIF4B Ser422 and Akt was analyzed as described in A. **C.** and **D.** eIF4B phosphorylation levels in A and B were quantitated by densitometry and normalized to total protein levels. The levels of eIF4B S422 phosphorylation are 100% at 0 h. Plotted are results from three independent experiments. Error bars represent SEM, *n* = 3 (*P < 0.05, **P < 0.01). **E.** and **F.** K562, NS2, or W44 cells were treated with 5 μM or 2 μM LY294002 for indicated time. The cell lysates were analyzed by Western blotting with indicated antibodies. **G.** and **H.** eIF4B phosphorylation levels in E and F were quantitated as described in C. **I.** experiments were performed as described in E. At 40 h, K562 cells treated with LY294002 were collected, washed with PBS and incubated with mixture of 5 μM LY294002 and 5 μM SMI-4a or with mixture of 5 μM LY294002 and vehicle for 8 h. Cells were analyzed for phosphorylation of eIF4B Ser422 by Western blotting.

Our previous studies have shown that Akt is upregulated in v-Abl-transformed cells derived from Pim triple knockout cells as compared to Pim wild-type counterparts [5]. In addition, experiments from other groups have also demonstrated that there exists a feedback regulation between Pim and Akt pathways in several cell types [5, 26, 27]. These findings prompted us to further address whether there is a cross-talk between STAT/Pim and PI3K/Akt/mTOR pathways and whether the two pathways co-operatively regulate eIF4B phosphorylation in Abl transformants. To this end, we blocked one pathway persistently, and then examined the activity of the other pathway. Interestingly, the activity of Akt was greatly upregulated by long time treatment of Bcr-Abl- or v-Abl-transformed cells with Pim inhibitor SMI-4a (Figure 2A and 2B). Importantly, although eIF4B Ser422 phosphorylation was at first suppressed by addition of SMI-4a, it was finally restored following upregulation of Akt activity after longer time treatment with the compound (Figure 2A–2D). To test whether this phenomenon was due to the loss of SMI-4a activity, we investigated the expression of c-Myc, since Pim inhibition can reduce c-Myc levels [28, 29]. As shown in Figure 2A and 2B, c-Myc expression remained suppressed during this period, suggesting that SMI-4a was functional.

Next, we asked whether STAT/Pim signaling could be activated by pharmacological inhibition of PI3K/Akt/mTOR pathway. Indeed, persistent blockage of PI3K by LY294002 resulted in activation of STAT5 and increased expression of Pim-1 (Figure 2E and 2F). Similarly, eIF4B Ser422 phosphorylation was inhibited by LY294002 treatment at short time point, but was then significantly restored following upregulation of Pim-1 expression induced by longer time treatment with LY294002 (Figure 2E–2H). Similar results were obtained from long time treatment with Akti-1/2 or rapamycin (Supplementary Figure S2A-S2H).

To corroborate whether the restoration of eIF4B phosphorylation resulted from enhanced activation of the other pathway due to persistent inhibition of one signaling pathway, we performed similar experiments as described in Figure 2A-2H except that the second inhibitor was added at late time points. As shown in Figure 2I, addition of Pim inhibitor SMI-4a at late time points prevented long term PI3K inhibition-induced eIF4B phosphorylation in K562 cells, suggesting that elevated Pim expression is responsible for the restoration of eIF4B phosphorylation. Inversely, addition of LY294002 at late time points also blocked long term Pim inhibition-induced eIF4B phosphorylation in the cells (Supplementary Figure S2I). Taken together, these data reveal that persistent inhibition of one signaling pathway results in upregulation of the other pathway, which can restore eIF4B Ser422 phosphorylation in Abl transformants.

### Simultaneous inhibition of Pim and PI3K/Akt/mTOR signaling reduces eIF4B phosphorylation more effectively than suppression of single pathway

**Figure 3:**
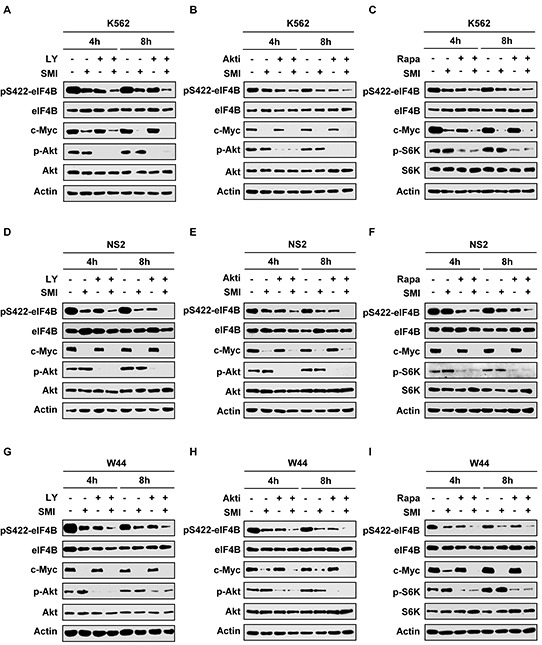
Combined inhibition of Pim and PI3K/Akt/mTOR signaling impairs eIF4B phosphorylation more effectively than suppressing one pathway alone **A.** K562 cells were treated with 12.5 μM SMI-4a or 10 μM LY294002 alone, or in combination for indicated time. Cell lysates were prepared and immunoblotted with indicated antibodies. **B.** and **C.** K562 cells were treated with 12.5 μM SMI-4a or 4 μM Akti-1/2 alone, or in combination (B); or 12.5 μM SMI-4a or 5 μM rapamycin alone, or in combination (C) for indicated time. Experiments were performed as described in A. **D–F.** NS2 cells were treated with/without 6.25 μM SMI-4a ± 5 μM LY294002 (D), 1.5 μM Akti-1/2 (E), or 2 μM rapamycin (F), for indicated time. Cell lysates were examined for eIF4B phosphorylation as described in A. **G–I.** experiments on W44 cells were performed as described in D. W44 cells were treated with/without 6.25 μM SMI-4a ± 5 μM LY294002 (G), 1.5 μM Akti-1/2 (H), or 2 μM rapamycin (I), for indicated time. Cell lysates were examined for levels of phospho-eIF4B (Ser422).

Our results presented above indicate that eIF4B is a convergent target of oncogenic Pim and PI3K/Akt/mTOR signaling in Abl transformants. Hence, we reasoned that combined inhibition of the two pathways would suppress eIF4B phosphorylation more effectively than inhibition of a signaling pathway. To test this concept, K562 cells were treated with SMI-4a and LY294002 simultaneously and eIF4B phosphorylation was examined at indicated time points (Figure 3A). As expected, the combined treatment displayed greater inhibition of eIF4B phosphorylation on Ser422 compared with the single treatment. Quantitative analysis of immunoblots by densitometry showed that eIF4B phosphorylation decreased more significantly by the combined inhibition of Pim and PI3K than the single inhibition (Supplementary Figure S3A). This observation was further confirmed by the combination using SMI-4a/Akti-1/2 or SMI-4a/rapamycin (Figure 3B and 3C and Supplementary Figure S3B and S3C). Similar results were obtained from experiments using SMI-4a/LY294002, SMI-4a/Akti-1/2, or SMI-4a/rapamycin to treat v-Abl transformants (Figure 3D–3I and Supplementary Figure S3D-S3I). These data suggest that combined inhibition of Pim and PI3K/Akt/mTOR pathways is required for efficient suppression of eIF4B phosphorylation in Bcr-Abl or v-Abl transformed cells.

### Combined pharmacological inhibition of Pim and PI3K/Akt/mTOR signaling has a synergistic effect on inducing apoptosis in Abl transformants

**Figure 4:**
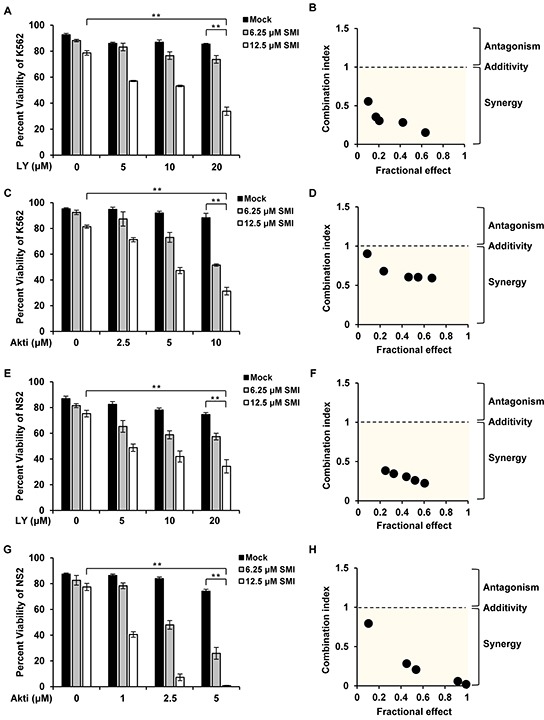
Combined inhibition of Pim and PI3K/Akt/mTOR pathways induces synergistic apoptosis **A.** K562 cells were treated with SMI-4a or LY294002 alone, or in combination at indicated concentrations for 48 h. Cells were harvested for Annexin V/PI staining of apoptotic cells. Values represent means ± SEM, *n* = 3 (**P < 0.01). **B.** The apoptosis of K562 cells in A were analyzed by Chou and Talalay method. Fractional effect denotes decrease of cell viability relative to control for apoptosis assay. Combination index (CI) value < 1 represents synergistic combination. **C.** experiments were performed as described in A. K562 cells were treated with SMI-4a or Akti-1/2 alone, or in combination at indicated concentrations for 48 h. Then cells were harvested for apoptosis analysis. **D.** the synergism of cell apoptosis in C was determined as described in B. **E–H.** NS2 cells were treated with SMI-4a or LY294002 alone, or in combination (E); with SMI-4a or Akti-1/2 alone, or in combination (G), at indicated concentrations for 24 h; cells were analyzed for apoptosis as described in A and CI values were calculated as described in B (F and H).

Phosphorylated eIF4B was previously shown to play a crucial role in regulating cell survival [4]. Having demonstrated that simultaneous inhibition of Pim and PI3K/Akt/mTOR signaling had a more profound inhibitory effect on eIF4B phosphorylation, next we studied whether the combined treatment influences the apoptosis of Abl transformants. Bcr-Abl-expressing K562 cells were treated with combination of SMI-4a and LY294002 at various concentrations for 48 h, and cell viability was examined by FACS after staining with Annexin V-FITC/PI (Figure 4A). We found that approximately 79% and 85% of K562 cells were viable after incubation with 12.5 μM SMI-4a or 20 μM LY294002 alone for 48 h, respectively, under our experimental condition. However, only 34% of the cells remained viable when treated with 12.5 μM SMI-4a plus 20 μM LY294002 under the same condition (Figure 4A). By the software analysis [30], we found that the combined index (CI) values for the combination of SMI-4a and LY294002 at different concentrations were all less than 1 (Figure 4B), indicating that this combination induces strong synergic apoptosis in K562 cells. Similar results were observed in K562 cells treated with SMI-4a and Akti-1/2 (Figure 4C and 4D), as well as SMI-4a and rapamycin (Supplementary Figure S4A and S4B).

To rule out the possibility that the higher concentrations of combined compounds or vehicle DMSO induced such synergy, we tested the effect of high concentrations of each inhibitor on apoptosis in Abl transformants (Supplementary Figure S4C-S4E). The results showed that treatment of the cells with high concentrations of single agent did not create a profound effect on inducing apoptosis as treatment with combined compounds did, suggesting that the synergy indeed resulted from the combinational inhibition of Pim and PI3K/Akt/mTOR pathways.

Next, the synergistic effect of the combined treatment on inducing apoptosis was tested in v-Abl-transformed cells. Strikingly, treatment with SMI-4a/LY294002 or SMI-4a/Akti-1/2 greatly sensitized NS2 cells to undergo apoptosis (Figure 4E–4H and Supplementary Figure S5A). Approximately 75% and 74% of NS2 cells were viable after 24 h incubation with 12.5 μM SMI-4a and 20 μM LY294002, respectively, but only 33% of cells remained viable after the combined treatment with 12.5 μM SMI-4a plus 20 μM LY294002 under the same condition (Figure 4E). The combinational treatment with SMI-4a/Akti-1/2 also induced synergistic apoptosis in NS2 cells (Figure 4G), as all CI values calculated were less than 1 (Figure 4F and 4H). Similar results were obtained from experiments using SMI-4a and rapamycin (Supplementary Figure S5B-S5F). Together, these data establish that the combined inhibition of Pim and PI3K/Akt/mTOR signaling has synergistic effect on inducing apoptosis in Abl transformants.

### Disruption of one pathway confers Abl transformants more sensitive to inhibition of the other pathway

**Figure 5:**
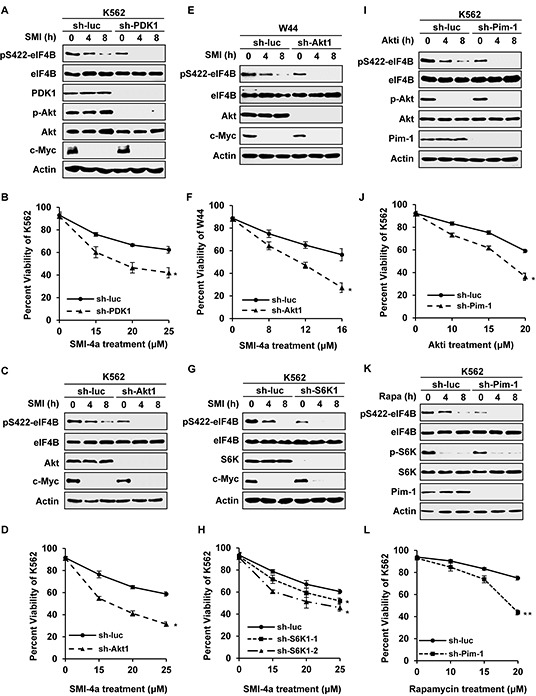
Silencing one pathway confers Abl transformants more sensitive to inhibition of the other pathway **A.** K562 cells stably expressing luciferase-specific shRNA or PDK1-specific shRNAs were treated with 15 μM SMI-4a for indicated time and immunoblotted for eIF4B phosphorylation. **B.** K562 cells in A were treated by indicated concentrations of SMI-4a for 48 h. Cells were harvested and apoptosis was displayed as percent viability using Annexin V staining followed by FACS. Values represent means ± SEM, *n* = 3 (*P < 0.05, **P < 0.01). **C–F.** K562 cells (C) or W44 cells (E) ectopically expressing shRNA targeting luciferase or Akt1 were treated with 15 μM SMI-4a (K562) or 8 μM SMI-4a (W44) in a time course, and analyzed by Western blotting as described in A. Cells were treated with indicated concentrations of SMI-4a for 48 h (K562) or 24 h (W44), and apoptosis of K562 (D) or W44 (F) was evaluated by FACS. **G.** and **H.** S6K1 knockdown or control K562 cells were incubated with 15 μM SMI-4a for indicated time and analyzed as described in A and B. **I–L.** K562 cells stably expressing luciferase-specific shRNA or Pim-1-specific shRNAs were treated with 10 μM Akti-1/2 (I) or 10 μM rapamyicn (K) for indicated time, and immunoblotted for eIF4B phosphorylation. Cells were incubated with Akti-1/2 for 48h (J) or rapamyicn for 36h (L) at indicated concentrations. Cell apoptosis was examined as described in B.

To further define the relationship between STAT/Pim and PI3K/Akt/mTOR pathways and their role in regulating eIF4B phosphorylation, genetic manipulation of individual signaling molecules in the two pathways was taken to examine the effects of suppressing these pathways on eIF4B phosphorylation and Abl transformant survival. For this, we generated Abl-transformed cells stably expressing shRNAs to either PDK1, Akt1, S6K1, or Pim-1. These cells were then treated with either Pim inhibitor or PI3K/Akt/mTOR inhibitors, followed by assessing eIF4B S422 phosphorylation and cell survival, as described above. We found that knockdown of PDK1 strengthened the inhibitory effect of Pim inhibitor SMI-4a on eIF4B S422 phosphorylation in K562 cells (Figure 5A and Supplementary Figure S6A). Using Annexin V/7-AAD staining followed by FACS, we observed that approximately 62% of control cells were viable after 48 h treatment with 25 μM SMI-4a, but only approximately 42% of PDK1 knockdown cells remained viable under the same condition (Figure 5B). Similar results were obtained from Akt1 knockdown K562 and W44 cells (Figure 5C-5F and Supplementary Figure S6B and S6C). Likewise, silencing Akt significantly enhanced the inhibitory effect of Pim inhibitor SMI-4a on eIF4B S422 phosphorylation in either K562 cell or W44 cells (Figure 5C and 5E and Supplementary Figure S6B and S6C), and sensitized Abl transformants to undergo apoptosis induced by the drug (Figure 5D and 5F). Moreover, silencing S6K1 also potentiated the inhibitory effect of Pim inhibitor SMI-4a on eIF4B Ser422 phosphorylation (Figure 5G and Supplementary Figure S6D), and enhanced SMI-4a-induced apoptosis of Abl transformants (Figure 5H).

In addition, we determined whether forced expression of Akt1 wild-type (WT) or the active form Akt1-E17K affects eIF4B phosphorylation and cell survival in response to SMI-4a treatment. Interestingly, overexpression of Akt1, especially Akt1-E17K, conferred high resistance to SMI-4a treatment, as evidenced by attenuated inhibition of eIF4B Ser422 phosphorylation and cell survival, compared to control (Supplementary Figure S6E-S6G).

On the other hand, Pim-1 knockdown cells were treated with PI3K/Akt/mTOR inhibitors, followed by the same analyses as described above. As shown in Figure 5I and Supplementary Figure S6H, eIF4B S422 phosphorylation in Pim-1 knockdown cells decreased significantly, compared with that in control cells after treatment with Akti-1/2. About 36% of Pim-1 knockdown cells were viable after treatment with 20 μM Akti-1/2 for 48 h, while approximately 60% of control cells remained viable under the same condition (Figure 5J), indicating that silencing Pim-1 sensitized Abl transformants to undergo apoptosis induced by Akti-1/2. Similar results were obtained from experiments using Pim-1 knockdown cells treated with rapamycin (Figure 5K and 5L and Supplementary Figure S6I). Together, these results suggest that disruption of one pathway alters the sensitivity of Abl transformants to inhibition of the other pathway by mediating eIF4B phosphorylation.

### Combined inhibition of Pim and PI3K/Akt/mTOR pathways suppresses the growth of K562 tumor engrafted in nude mice in a synergistic manner

**Figure 6:**
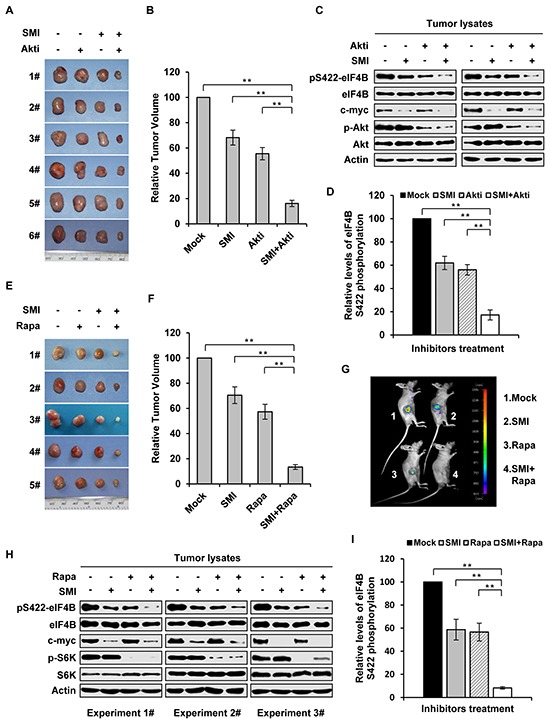
Combined inhibition of Pim and PI3K/Akt/mTOR pathways suppresses the growth of K562 tumor engrafted in nude mice in a synergistic manner **A.** nude mice carrying K562 xenografts were treated with SMI-4a (50 mg/kg) alone, Akti-1/2 (80 mg/kg) alone, their combination, or vehicle control every other day. Tumors were excised at 8 h after the last treatment. Shown are representatives from six independent experiments. **B.** plotted are relative volumes of tumors in A. The average volume of vehicle control was set as 100%. Error bars, SEM, *n* = 24 (**P < 0.01). **C.** eIF4B Ser422 phosphorylation in representative tumors in A was examined by immunoblotting. **D.** levels of phosphorylated eIF4B in C were quantitated by densitometry, and normalized to total eIF4B levels. The levels of Ser422 phosphorylation of Mock group were 100%. Plotted are results from three independent experiments. Error bars represent SEM, *n* = 3 (**P < 0.01). **E.** nude mice bearing GFP-expressing K562 xenografts were treated with SMI-4a (50 mg/kg) alone, rapamycin (10 mg/kg) alone, combined treatment, or vehicle control every other day. Tumors were excised at 8 hours after the last treatment. Shown are representatives from five independent experiments. **F.** shown are relative volumes of tumors in E. The average volume of vehicle control was set as 100%. Error bars, SEM, *n* = 20 (**P < 0.01). **G.** over a 21-day period after xenograft inoculation, tumors under indicated treatments were measured by bioluminescent imaging. Shown are representative images from at least three independent experiments with similar results. **H.** tumors in E were examined by immunoblotting with indicated antibodies. **I.** levels of phosphorylated eIF4B in H were quantitated by densitometry, and normalized to total eIF4B levels as described in D.

In an attempt to explore the feasibility of this combined therapeutic strategy for treatment of Abl-mediated tumorigenesis, we tested the efficacy of inhibiting both Pim and PI3K/Akt/mTOR pathways in a mouse xenograft model. Each mouse was inoculated subcutaneously with the same amount of K562 cells, and administration of either single agent (SMI-4a or Akti-1/2), combination (SMI-4a and Akti-1/2), or mock was then conducted on day 11 after inoculation of the cells. On the 29^th^ day after inoculation, tumors were removed from mice and their volumes were measured as previously described [4]. We observed that the combined treatment with 50 mg/kg of SMI-4a and 80 mg/kg of Akti-1/2 inhibited Abl-mediated tumor growth more potently than the treatment with either 50 mg/kg of SMI-4a or 80 mg/kg of Akti-1/2 alone (Figure 6A and 6B). SMI-4a in combination with Akti-1/2 suppressed the tumor growth in a synergistic manner, as determined by the formula AB/C < A/C × B/C described previously [31] and in Materials and Methods (AB/C = 0.162, A/C = 0.682, B/C = 0.555, A/C × B/C =0.379). Moreover, combined treatment with SMI-4a and Akti-1/2 inhibited eIF4B Ser422 phosphorylation more significantly than that with SMI-4a or Akti-1/2 alone (Figure 6C and 6D).

In addition, similar experiments using SMI-4a and rapamycin were performed. As shown in Figure 6E and 6F, combined treatment of mice with 50 mg/kg of SMI-4a and 10 mg/kg of rapamycin also suppressed the tumor growth in a synergistic manner (AB/C = 0.134, A/C = 0.705, B/C = 0.573, A/C × B/C =0.404). These results were further confirmed by bioluminescent imaging (Figure 6G). Furthermore, Western blotting analysis of tumor lysates showed that eIF4B Ser422 phosphorylation was more significantly reduced by the combined inhibition of Pim and mTOR as compared to the single inhibitions (Figure 6H and 6I).

### Decreased phosphorylation of eIF4B on Ser422 is responsible for synergic inhibitory effect on cell survival by combined blockage of Pim and PI3K/Akt/mTOR pathways

**Figure 7:**
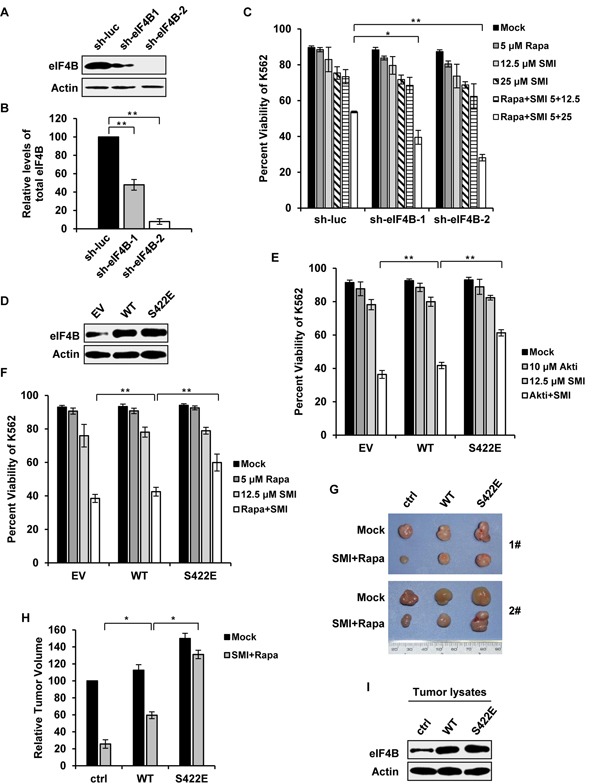
Altered phosphorylation of eIF4B on Ser422 is responsible for synergic inhibition of cell survival by combined blockage of Pim and PI3K/Akt/mTOR pathways **A.** interference efficiency of shRNA-based knockdown of eIF4B in K562 cells was determined by Western blotting. shRNA targeting luciferase (sh-luc) served as a control. **B.** protein levels of eIF4B in A were quantitated by densitometry and normalized to β-actin. Plotted are the average levels from three independent experiments. The error bars represent the SEM (**P < 0.01). **C.** K562 cells in A were treated with SMI-4a alone, rapamycin alone, or their combination at indicated concentrations. After 36 h, the cells were assessed for apoptotic fraction. Values represent means ± SEM, *n* = 3 (*P < 0.05, **P < 0.01). **D.** K562 cells ectopically expressing empty vector, eIF4B-WT or eIF4B-S422E mutant were examined by Western blotting. **E.** cells in D were treated with 12.5 μM SMI-4a alone, 10 μM Akti-1/2 alone, or their combination. After 48 h, cells were harvested for Annexin V staining as described in C. **F.** cells in D were treated with 12.5 μM SMI-4a alone, 5 μM rapamycin alone, or their combination for 48 h. Apoptosis was examined as described in C. **G.** K562 cells expressing vector, eIF4B-WT or eIF4B-S422E mutant were transplanted into nude mice. At the 11^th^ day after inoculation, mice bearing xenografts were treated with mock or combination of 50 mg/kg SMI-4a and 10 mg/kg rapamycin each other day. At the 29^th^ day mice were sacrificed. Shown are representative tumors under mock or drug administration. **H.** the relative tumor volumes were displayed as described in Figure 6B. The average volume of tumor (empty vector) with mock treatment was set as 100%. Error bars, SEM, *n* = 18 (*P < 0.05). **I.** tumors in G were immunoblotted with indicated antibodies. Shown are representative results from three independent experiments.

Having revealed that eIF4B is a common substrate of Pim kinase and PI3K/Akt/mTOR pathways, we hypothesized that eIF4B phosphorylation on Ser422 might be critical for synergic apoptosis of Abl transformants induced by the combinational treatment. To test this hypothesis, firstly we generated K562 cells stably expressing shRNAs targeting eIF4B (Figure 7A and 7B). We found that compared to control, disruption of eIF4B expression significantly sensitized K562 leukemic cells to undergo apoptosis induced by combined treatment with SMI-4a and rapamycin (Figure 7C), suggesting the functional significance of eIF4B in the regulation of Abl-transformed cell survival by Pim and PI3K/Akt/mTOR pathways. Furthermore, we generated stable K562 cells expressing either eIF4B-WT, its phosphomimetic mutant eIF4B-S422E, or empty vector control (Figure 7D). When the cells were treated with 12.5 μM SMI-4a and 10 μM Akti-1/2 for 48 h, the cell viability in the control, eIF4B-WT, and eIF4E-S422E cells was reduced to approximately 36%, 42% and 61%, respectively (Figure 7E). Similar results were obtained from experiments using combined treatment with SMI-4a and rapamycin (Figure 7F). The results suggest that the synergic effect of combined inhibition of Pim and Akt/mTOR on cell survival could be substantially attenuated by forcing expression of eIF4B S422E mutant.

To confirm the importance of eIF4B Ser422 phosphorylation in Abl-mediated tumorigenesis, we further performed xenograft experiments using K562 cells stably expressing either eIF4B-WT, eIF4B-S422E, or empty vector. Mice were inoculated with equal amount of each cell type and treated with the combination of SMI-4a and rapamycin as described above. Indeed, the synergic inhibition of tumor growth by the combined treatment was greatly compromised by the expression of eIF4B-S422E in K562 cells (Figure 7G and 7H). The ectopic expression of eIF4B proteins in tumors was confirmed by Western blotting (Figure 7I). Together, these data indicate that eIF4B functions downstream of STAT/Pim and PI3K/Akt/mTOR signaling in Abl transformants, and Ser422 phosphorylation is associated with synergic inhibitory effects on cell survival and tumor growth induced by combined blockage of Pim and PI3K/Akt/mTOR pathways.

## DISCUSSION

Abl-mediated tumorigenesis of hematopoietic cells involves various complex mechanisms including dysregulation of gene transcription and alterations in protein synthesis important for cell growth and apoptosis [9, 10]. In this study, we show that eIF4B is a key convergent substrate of Pim and PI3K/Akt/mTOR pathways in Abl-transformed cells. Selective inhibition or silencing of Pim (with SMI-4a), PI3K (with LY294002), Akt (with Akti-1/2), or mTOR (with rapamycin) was able, to some extent, to impair eIF4B Ser422 phosphorylation in the cells. Although both JAK/STAT/Pim and PIK3/Akt/mTOR pathways are involved in mediating phosphorylation of eIF4B on Ser422 in Abl transformants, Pim kinases may contribute more to this process [4]. In normal cells, coordinated regulation of eIF4B phosphorylation by multiple signaling pathways may be required for efficient cell proliferation and growth. However, this redundant mechanism of eIF4B activation can be hijacked by cancer cells to evade pharmacological treatment and develop drug tolerance, thus resulting in short-lived single agent targeted tumor therapy [30–36]. Indeed, our data reveal that single targeting Pim or PI3K pathway displayed limited inhibitory effect on survival and growth of Abl-transformed cells, especially after long-term treatment with single compound. Furthermore, such failure was found to result from the parallel redundant pathway that provides enough ability to restore eIF4B phosphorylation. Thus, the combined treatment that abrogates the Pim and PI3K/Akt/mTOR pathways is able to impair the phosphorylation of eIF4B more effectively, leading to profound inhibitory effect on Abl-mediated tumorigenesis in a synergistic manner.

It has been shown that components of the translation initiation machinery are often deregulated in various cancer cells [4, 8–11, 15, 21, 30, 37]. For example, aberrant activation of eukaryotic translation initiation factor 4E (eIF4E) has been shown to be critical for tumorigenesis of a number of cancers including lymphomas, angiosarcomas, lung carcinomas, and hepatomas [38]. Elevated expression of eukaryotic translation initiation factor 3b (eIF3b) correlates to human bladder and prostate cancer progression [39]. In addition, oncogenic Pim-2 kinase has also been shown to modulate the translation initiation repressor eIF4E-binding protein 1 (4E-BP1) [35, 40]. It has also been found that there exists a convergent crosstalk between ERK and Akt signaling pathways that converge on 4E-BP1 to control its activity [41–43]. Thus, 4E-BP1 controls tumor growth through integrating the functions of Akt and ERK signaling pathways [41–43]. Previous experiments have demonstrated that there co-exists activation of oncogenic Pim and Akt pathways in Abl-transformed cells [4–6]. Here we observed that persistent inhibition of one signaling pathway strikingly induced the activation of the other pathway, causing the Abl-expressing leukemic cells to become dependent on the latter. These data reveal the molecular basis for the limited activity of the single Pim or Akt inhibitor in Abl-transformed cells and the profound synergy observed with combined inhibition of the dual pathways. In this study, for the first time, we provide evidence that eIF4B is a convergent target of Pim and Akt pathways in Abl transformants. Reduced phosphorylation of eIF4B on Ser422 correlates with the synergistic anticancer activity of the combined treatment to block the two pathways. This is strongly supported by the observation that ectopic expression of eIF4B phosphomimetic mutant S422E dramatically attenuated the synergic effects of the combined treatment. Taken together, our data indicate that eIF4B integrates the functions of Pim and Akt signaling pathways to stimulate Abl-mediated tumorigenesis.

Accumulating evidence suggests that redundant activation of multiple signaling pathways may converge on common targets that integrate their functions in cancers. For example, BCL2-associated agonist of cell death (BAD) functions as a shared target of Akt and ERK signaling in phosphatase and tensin homolog (PTEN) deficient tumor cells [32]. Phosphorylation of BAD by either Akt or ERK is sufficient to suppress the activity of BAD. However, only inhibiting the two pathways simultaneously can activate BAD and induce apoptosis in tumor cells [32]. On the other hand, occurrence of activation of multiple pathways is a major cause of drug resistance in tumor cells. For example, melanoma cells display enhanced activation of PI3K signaling after treatment with BRAF inhibitor, resulting in tumor cell resistance to the drug challenges [33]. In addition to ERK signaling, activation of other pathways is also responsible for the limited effect of PI3K/Akt/mTOR inhibitors on tumorigenesis [31, 35, 36]. In lymphoma, overexpression of Pim kinases results in resistance to mTOR inhibitors [35]. In metastatic breast cancer, genetic or pharmacological inhibition of JAK2/STAT5 makes cells more sensitive to PI3K/Akt/mTOR inhibitors [31]. These studies suggest that growth of tumors carrying oncogenes activating multiple pathways is not dependent on a single signaling pathway. Therefore, it is important to identify the common targets that integrate the functions of multiple pathways. The mechanism underlying the redundant regulation still remains unclear. It might be explained partially by the polyclonal and polygenic nature of cancer cells [36]. Some investigations suggest that such redundancy may increase fitness of cancer cells in certain unfavorable environments [30, 44, 45]. Consistent with this hypothesis, our data show that genetic disruption of either Pim or Akt pathway sensitized Abl transformants to undergo apoptosis induced by inhibition of the other, and inhibition of both was required to efficiently suppress Abl oncogenicity. Previous studies revealed that silencing eIF4B expression is sufficient to inhibit Abl transformation [4]. Here we show that eIF4B integrates the functions of oncogenic Pim and Akt signaling pathways. These findings suggest that eIF4B may be a candidate for targeted therapy against the malignancies in which both Pim and Akt pathways are activated. However, further studies are needed to determine the clinical significance of directly targeting eIF4B in CML patient samples and other cancers.

## MATERIALS AND METHODS

### Cell lines, cell culture and Western blotting

Cell lines K562 and 293T were purchased from American Type Culture Collection (ATCC) and cultured in RPMI-1640 or Dulbecco's Modified Eagle Medium supplemented with 10% fetal bovine serum (FBS) as described previously [4]. The v-Abl transformed mice pre-B cell lines NS2 and W44 were generated and cultured as previously described [1]. These cell lines were characterized in our laboratory as CD10^+^/CD19^+^ pre-B cells. Abl transformants stably expressing short hairpin RNA (shRNA) targeting human or mouse PDK1, Akt1, S6K1, Pim-1, eIF4B were generated by infection with lentiviruses as described previously [4, 46, 47]. Paired shRNA sequences were designed and listed in the Supplementary Materials and Methods section. Western blotting was performed as described previously [4, 5]. Briefly, total cell extracts were separated by SDS-polyacrylamide gel electrophoresis, transferred onto nitrocellulose membrane, and probed with indicated antibodies. Where indicated, immunoblotting signals were quantified by densitometry [4].

### Antibodies and reagents

The following antibodies were used in this study: anti-phospho-eIF4B (Ser422) (SC-293101, Santa Cruz Biotechnology), anti-eIF4B (SC-376062, Santa Cruz Biotechnology), anti-phospho-STAT5 (Tyr694) (9359L, Cell Signaling), anti-STAT5 (9358S, Cell Signaling), and ani-PDK1 (3062S, Cell Signaling). The inhibitors were purchased as follows: Pim kinase inhibitor SMI-4a (526523, Merck), PI3K inhibitor LY294002 (L9908, Sigma), Akt inhibitor Akti-1/2 (124018, Merck), and mTOR inhibitor rapamycin (SC-3504A, Santa Cruz). All other antibodies and reagents were obtained as previously described [4, 5, 48].

### Cell apoptosis and proliferation assays

Apoptosis assay was performed as previously described [4, 5]. Briefly, cells were cultured with certain inhibitors for indicated time. Then cells were stained with 2.5 μg/ml Annexin V-FITC and 1 μg/ml PI. For cells overexpressing or silencing given genes, samples were stained with 2.5 μg/ml Annexin V-APC and 1 μg/ml 7-AAD. Samples were examined by fluorescence-activated cell sorter (FACS) (BD Bioscience) as previously described [5] and data were analyzed by FCS Express V3 Software (De Novo Software). For experiments using combined treatment, the synergism was evaluated by the combination index (CI) which was calculated using Chou-Talalay method by CompuSyn software (ComboSyn, Inc.) [49, 50]. Briefly, CI value < 1 represents synergistic effect, CI values = 1 additive effect, and CIs > 1 antagonistic effect.

### Animal experiments

K562 cells (5 × 10^6^ cells per mouse) were transplanted subcutaneously into Balb/c nude mice (5-6 weeks old) (Vital River, Beijing, China). Starting from the 11^th^ day after inoculation, mice were treated with inhibitors or mock every other day and tumor volume was monitored simultaneously. SMI-4a (50 mg/kg) and Akti-1/2 (80 mg/kg) were administered orally every other day. Rapamycin (10 mg/kg) was injected intraperitoneally every two days. At 8 h after the 10^th^ treatment, mice were sacrificed and tumors were removed for further analysis. Tumor sizes were analyzed for synergism according to the formula AB/C < A/C × B/C, where A was the tumor volume treated with inhibitor 1, B was the tumor volume treated with inhibitor 2, AB was the tumor volume treated with inhibitors 1 and 2, and C was the tumor volume of control group [31]. Bioluminescent imaging was performed at day 21 after inoculation, and photography and image quantification were carried out as previously described [7].

### DNA construction

Short hairpin RNA (shRNA) expressing constructs were generated as previously described [4]. The shRNA sequences targeting human eIF4B, human Pim-1 and human/mouse S6K1, were described previously [4]. The shRNA sequences are shown as follows:

human Akt1: 5′-CGCGTGACCATGAACGAGTTT-3′ and 5′-CGAGTTTGAGTACCTGAAGCT-3′; mouse Akt1: 5′-CGTGTGACCATGAACGAGTTT-3′ and 5′-CGAGTT TGAGTACCTGAAGCT-3′); human PDK1: 5′-CAAAGT TCTGAAAGGTGAAAT-3′ and 5′-GCAGCAACATAGA GCAGTACA-3′; mouse PDK1: 5′-GAATTTGCACCAG CAGACA-3′ and 5′-GGCTAGAGATCTTGTGGAA-3′.

### Ethics statement

The mouse experimental design and protocols used in this study were approved by “the Regulation of the Institute of Microbiology, Chinese Academy of Sciences of Research Ethics Committee” (Permit Number: PZIMCAS2013008). All mouse experimental procedures were performed in accordance with the Regulations for the Administration of Affairs Concerning Experimental Animals approved by the State Council of People's Republic of China.

### Statistical analysis

Data were displayed as mean values ± standard error (mean ± SE) of at least three independent experiments. Statistical significance was determined by Student's t-test and the P values < 0.05 was considered to be significant.

## SUPPLEMENTARY FIGURES


