# Supplementary material for: eIF4B is a convergent target and critical effector of oncogenic Pim and PI3K/Akt/mTOR signaling pathways in Abl transformants
Source: Oncotarget. 2016 Feb 3;7(9):10073–89. doi: 10.18632/oncotarget.7164 (PMC4891105; doi:10.18632/oncotarget.7164)
Supplement: Supplementary file 1 [file oncotarget-07-10073-s001.pdf]

SUPPLEMENTARY FIGURES

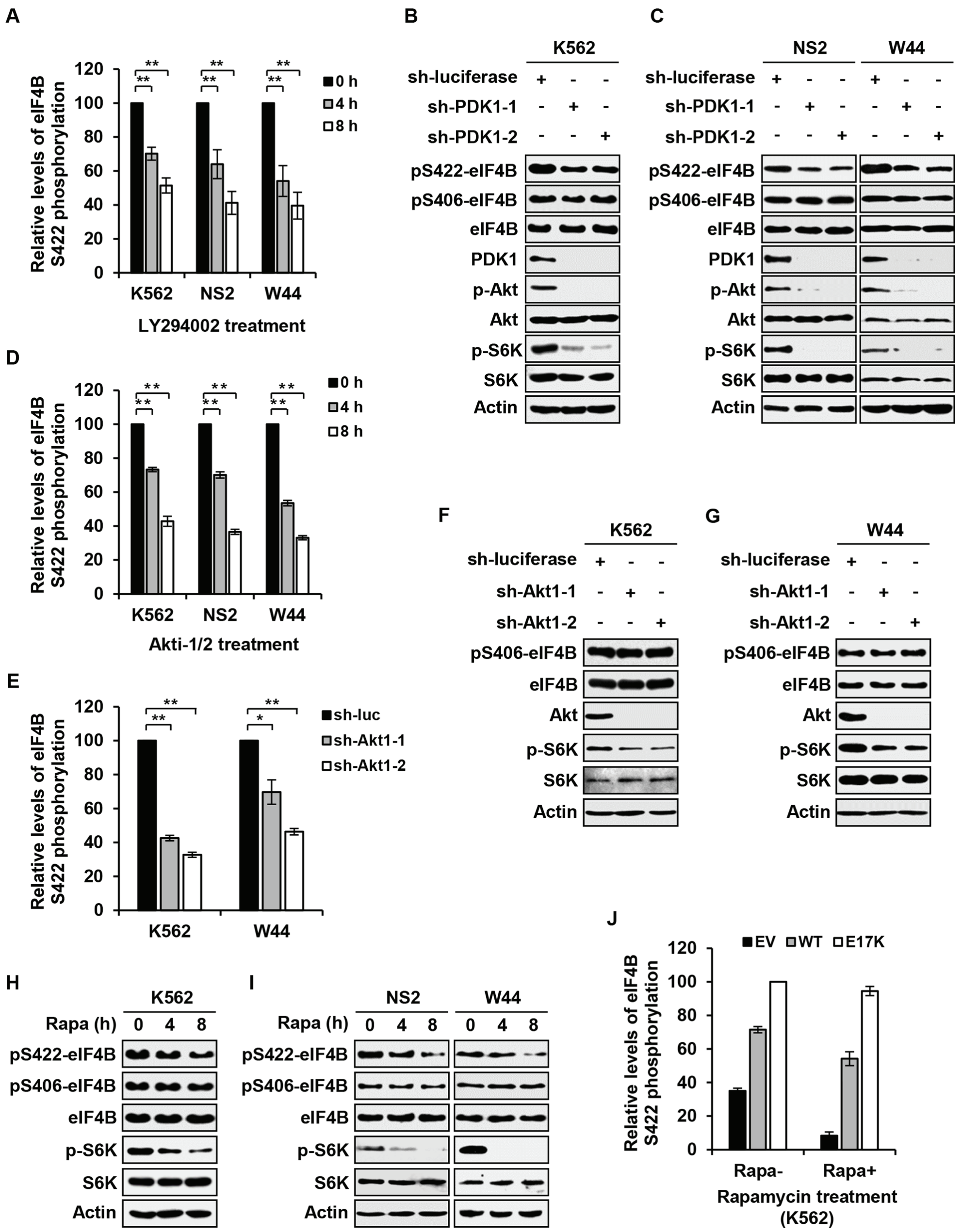

Supplementary Figure S1: eIF4B S422 phosphorylation is regulated by PI3K/Akt/mTOR pathway in Bcr-Abl and v-Abl transformants. A. eIF4B Ser422 phosphorylation levels in Figure 1A and B were quantitated by densitometry and normalized to total protein levels. The levels of eIF4B S422 phosphorylation were set to 100% at 0 hour. Plotted are results from three independent experiments. Error bars represent SEM, $n = 3$ (*$P < 0.05$, **$P < 0.01$). **B.** and **C.** Bcr-Abl-transformed cells (B) or v-Abl-transformed cells (C) stably expressing luciferase-specific shRNA (sh-luc) or PDK1-specific shRNAs were analyzed by Western blotting with indicated antibodies. **D.** eIF4B Ser422 phosphorylation levels in Figure 1D and E were quantitated as described in A. **E.** eIF4B Ser422 phosphorylation levels in Figure 1F and G were quantitated as described in A. **F.** and **G.** Abl transformed cells in Figure 1F and G were analyzed for eIF4B Ser406 phosphorylation levels with indicated antibodies. **H.** and **I.** K562 or v-Abl⁺ cells (NS2 and W44) were treated with 5 μM (H) or 2 μM (I) rapamycin for indicated time. Shown are immunoblots probed with indicated antibodies. **J.** eIF4B phosphorylation levels in Figure 1J were quantitated and normalized as described in A. The highest level of eIF4B Ser422 phosphorylation was set to 100%. Plotted are average results from three independent experiments. Error bars represent SEM, $n = 3$.

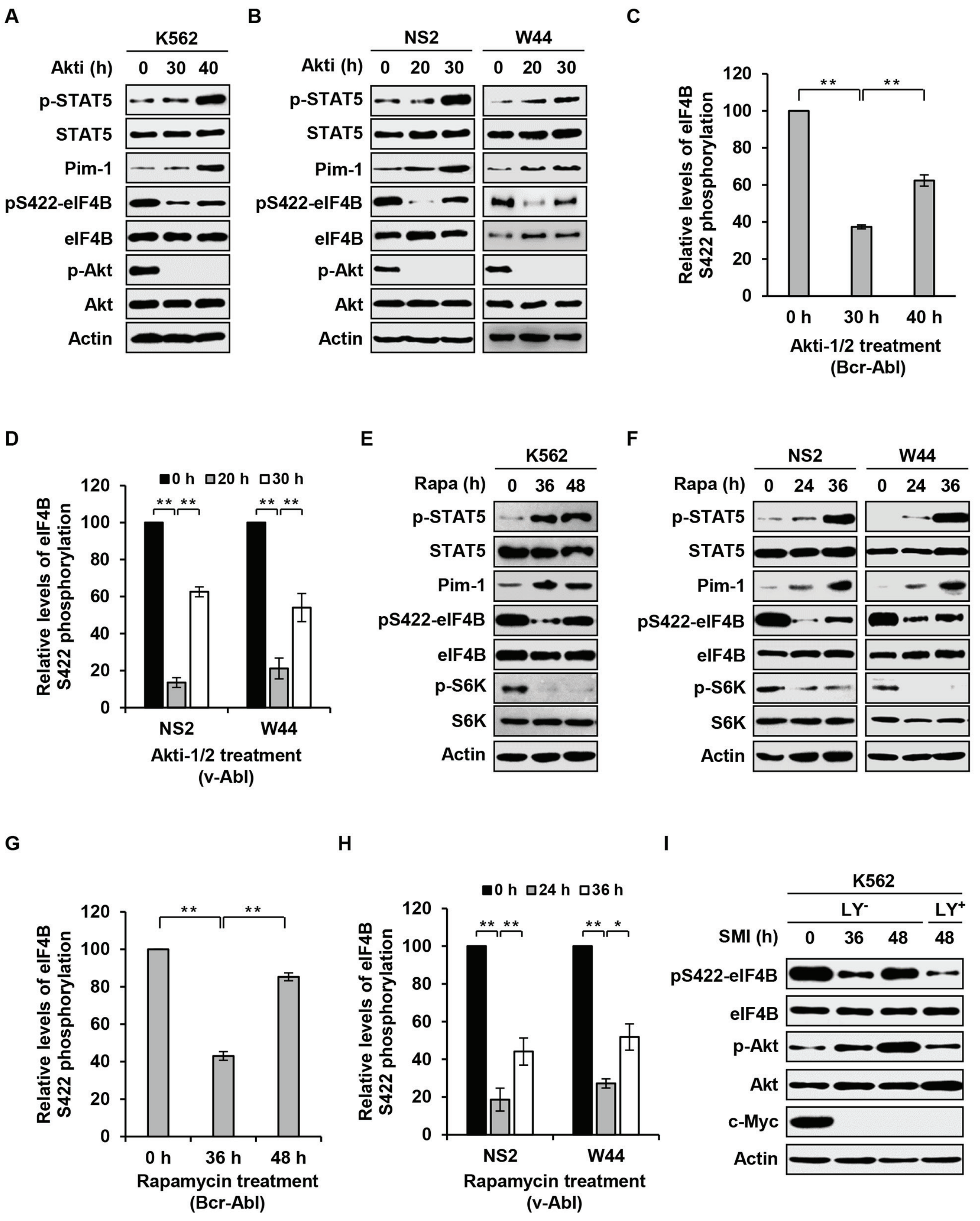

Supplementary Figure S2: Long time inhibiting one pathway in Abl transformants activates the other pathway. A. K562 cells were treated with 1.5 μM Akti-1/2 for indicated time. Cells were examined by Western blotting with indicated antibodies. **B.** NS2 and W44 cells were treated with 800 nM Akti-1/2 for indicated time and analyzed as described in A. **C.** and **D.** shown is quantification of eIF4B S422 phosphorylation levels in A and B. Plotted are average results from three independent experiments. The levels of eIF4B S422 phosphorylation were set to 100% at 0 hour. Error bars, SEM, $n = 3$ (*$P < 0.05$, **$P < 0.01$). **E.** and **F.** Bcr-Abl$^+$ cells (E) or v-Abl$^+$ cells (F) were treated with 2 μM (E) or 1μM (F) rapamycin for indicated time and examined as described in A. **G.** and **H.** shown is quantification of eIF4B S422 phosphorylation levels in E and F as described in C. **I.** K562 cells were treated with 5 μM SMI-4a as described in Figure 2A. At 40 h, K562 cells treated with SMI-4a were collected, washed with PBS and incubated with mixture of 5 μM SMI-4a and 5 μM LY294002 or with mixture of 5 μM SMI-4a and vehicle for 8 h. Shown is an immunoblot probed with indicated antibodies.

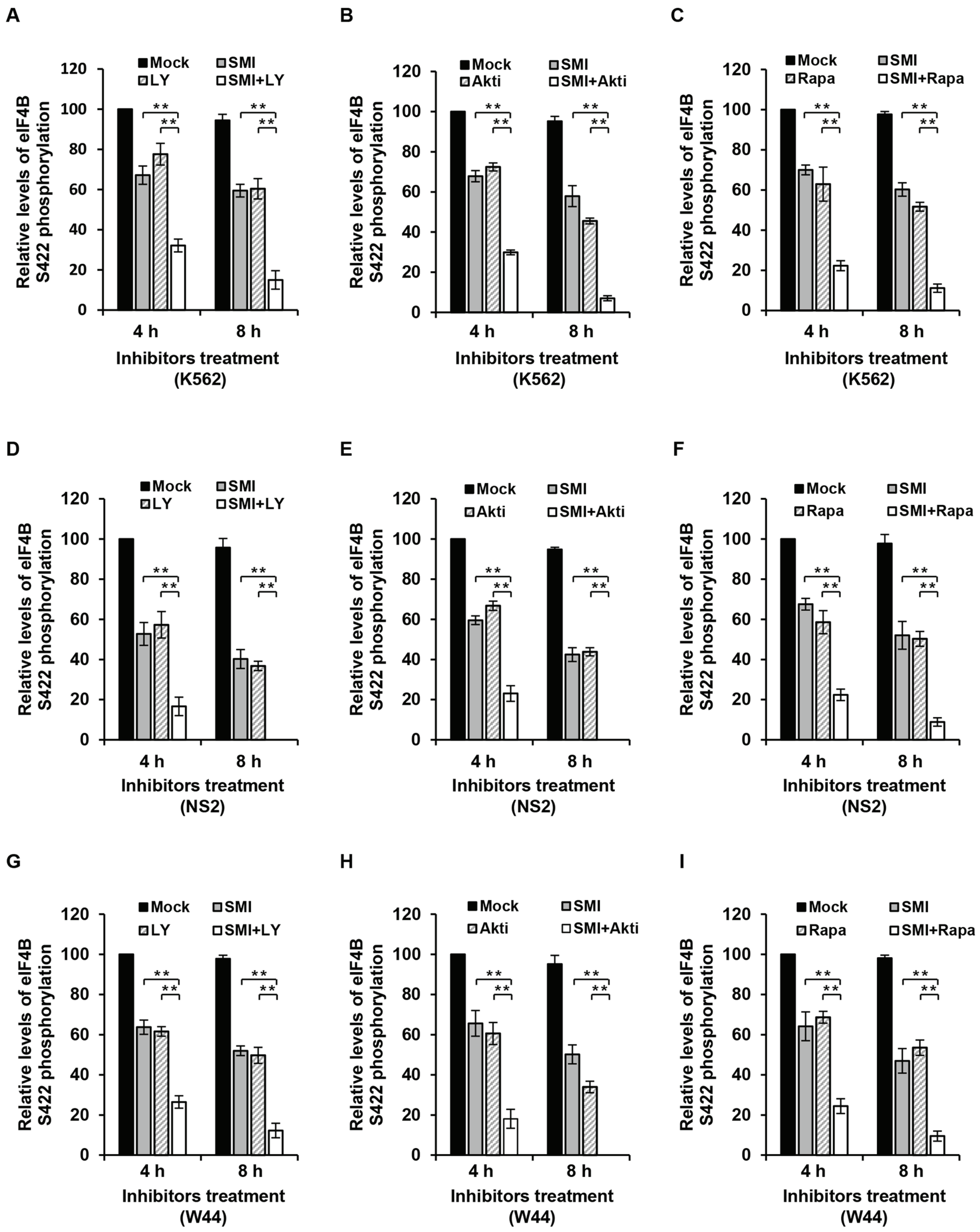

Supplementary Figure S3: Relative levels of eIF4B phosphorylation on S422 in cells under combined or single treatment. A–I. shown are the densitometric results of eIF4B S422 phosphorylation levels in Figure 3A-I. The relative phospho-eIF4B levels were normalized to total eIF4B levels, and S422 phosphorylation of mock group at 4 h was set as 100%. Plotted are average results from three independent experiments. Values are displayed as means ± SEM, $n = 3$ (**$P < 0.01$).

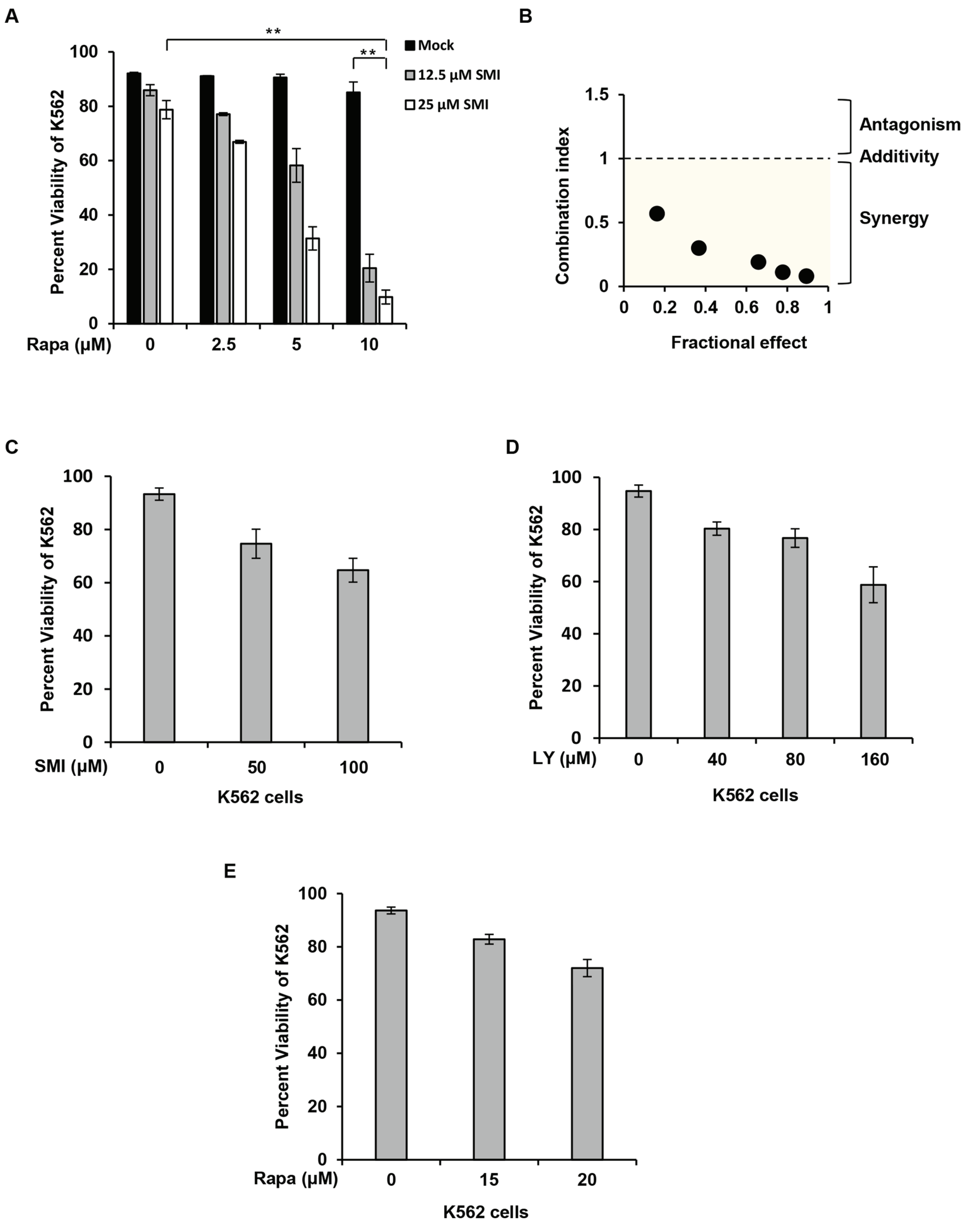

Supplementary Figure S4: Profound apoptosis is induced by combined drug treatment, but not by high concentrations of single inhibitor. **A.** and **B.** K562 cells were treated with SMI-4a alone, rapamycin alone, or their combination at indicated concentrations for 36 h. Cells were examined for apoptosis as described in Figure 4A. CI values were calculated as described in Figure 4B. **C.** K562 cells were treated with high concentrations of SMI-4a for 36 h. Cells were then examined for apoptosis as described in A. **D.** and **E.** K562 cells were cultured with high concentrations of LY294002 (D) or rapamycin (E) for 48 h or 36 h respectively. Apoptosis were assessed as described in C.

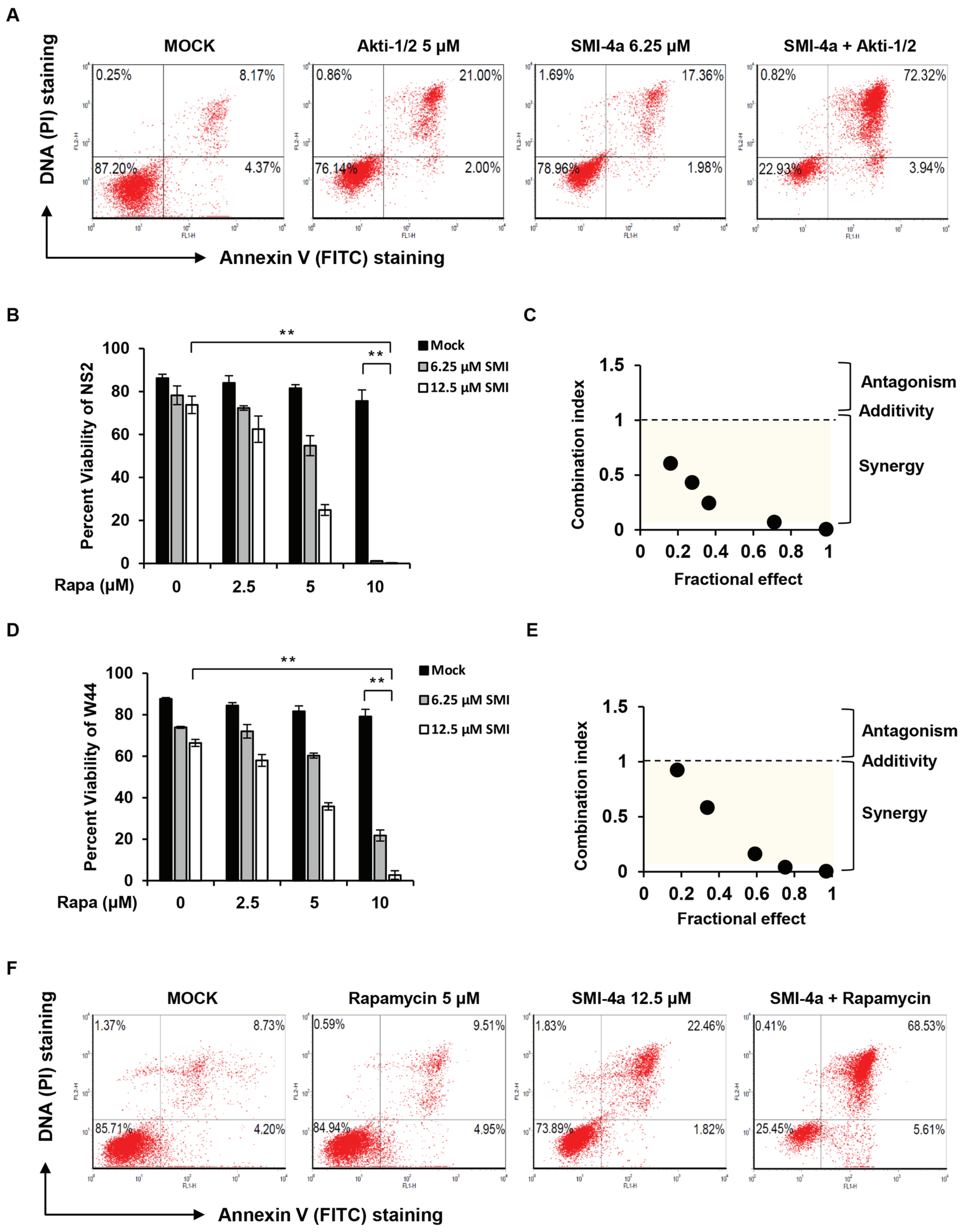

Supplementary Figure S5: Simultaneously inhibiting Pim and PI3K/Akt/mTOR signaling results in synergic inhibition on v-Abl transformant survival. A. as described in Figure 4G, NS2 cells were treated with SMI-4a, Akti-1/2, alone or in combination for 24 h. Cell apoptosis were examined by FACS. Shown are representative images from at least three independent experiments. **B–E.** v-Abl transformed cells NS2 (B) and W44 (D) were treated with SMI-4a, rapamycin, alone or in combination at indicated concentrations for 24 h. Apoptosis was also assessed by FACS as described in Figure 4A. The CIs were calculated (C and E). **F.** shown are representative images from at least three independent experiments testing NS2 cells in B.

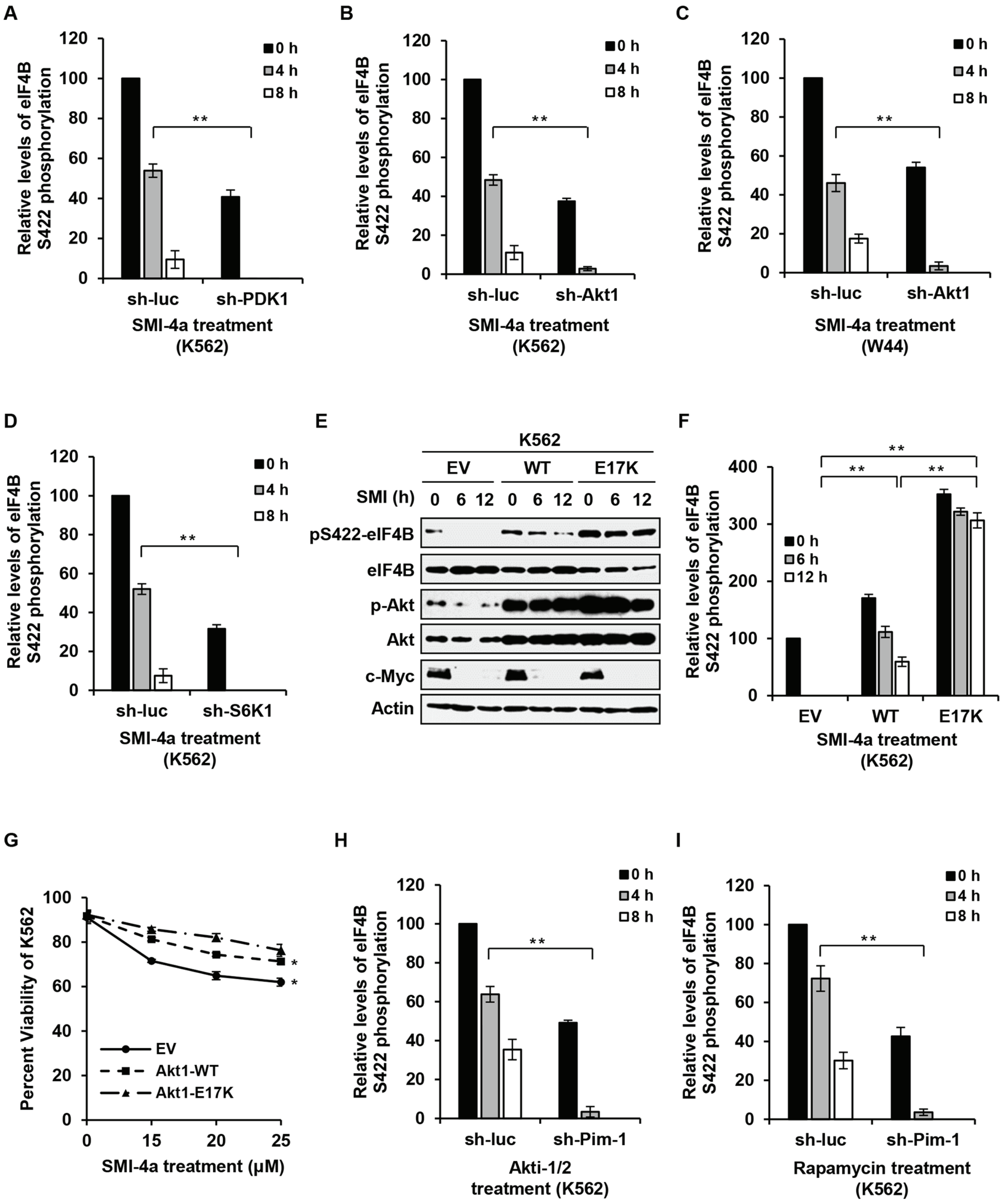

Supplementary Figure S6: eIF4B S422 phosphorylation is regulated by Pim and PI3K/Akt/mTOR in Abl transformants and combined inhibition of two pathways causes profound apoptosis. A–D. shown are the densitometric data of eIF4B S422 phosphorylation levels in Figure 5A, 5C, 5E, and 5G. The relative phospho-eIF4B levels were normalized to total eIF4B levels, and S422 phosphorylation of control group at 0 h was set as 100%. Plotted are average results from three independent experiments. Values are displayed as means ± SEM, n = 3 (**$P < 0.01$). **E.** K562 cells ectopically expressing empty vector (EV), Akt1-WT or Akt1-E17K were treated with 15 μM SMI-4a for indicated time and probed with indicated antibodies in Western blotting. **F.** shown are the densitometric data of eIF4B S422 phosphorylation levels in E. **G.** cells in E were treated as described in Figure 5B and cell apoptosis was evaluated by FACS. Values represent means ± SEM, n = 3 (*$P < 0.05$). **H.** and **I.** shown are quantitative data of eIF4B S422 phosphorylation levels in Figure 5I and 5K. Plotted are the results from at least three independent experiments. Values are displayed as means ± SEM, n = 3 (**$P < 0.01$).
